# Impact of Silanized Nanographene Oxide Concentrations in Different Primers on Bonding Durability between Resin Cement and Zirconia

**DOI:** 10.1055/s-0044-1795126

**Published:** 2025-03-10

**Authors:** Ahmed Q. Mahmoud, Tarek Ahmed Soliman, Tarek A. Elkhooly, Asmaa Harhash, El-Sayed Gad Eid

**Affiliations:** 1 Department of Dental Biomaterials, Faculty of Dentistry, Egyptian Russian University, Cairo Governorate, Egypt; 2 Department of Dental Biomaterials, Faculty of Dentistry, Mansoura University, Mansoura, Egypt; 3 Department of Conservative Dental Sciences, College of Dentistry, Prince Sattam Bin Abdulaziz University, Al-Kharj, Saudi Arabia; 4 Department of Refractories, Ceramics, and Building Materials, National Research Centre, Dokki, Giza, Egypt; 5 Nanomedicine Research Unit, Faculty of Medicine, Delta University for Science and Technology, Gamasa, Egypt; 6 Department of Restorative Dentistry, College of Dentistry, University of Science and Technology of Fujairah, Fujairah, United Arab Emirates

**Keywords:** zirconia, silanized nanographene oxide, primers, resin cement, durability, bond strength, wettability

## Abstract

**Objectives** Zirconia (ZrO _2_ ) has been used in dental restorations due to its increased mechanical properties, biocompatibility, low degree of bacterial adhesion, and acceptable optical properties. One of the major drawbacks of ZrO _2_ is its short-term durable bond with resin cement. The objective of this study was to evaluate the effect of different primers embedded with silanized nanographene oxide (SGO) sheets on the wettability of ZrO _2_ surface and bond strength durability between resin cement and ZrO _2_ .

**Materials and Methods** Four hundred ZrO _2_ specimens were divided into four main groups as each group had 100 specimens according to the type of the primer: rely X ceramic primer (Group I), monobond N primer (Group II), monobond plus primer (Group III), and Z prime plus primer (ZP, Group IV). Each main group was subdivided into five subgroups according to SGO concentrations by weight blended into primers: (1) 0% (control), (2) 0.1%, (3) 0.3%, (4) 0.6%, and (5) 0.9% as each subgroup had 20 specimens. Immediate shear bond strength (SBS) test was done for half of the specimens per each subgroup (10 specimens) by universal testing machine, the other half of the specimens per each subgroup (10 specimens) were exposed to thermocycling for 10,000 cycles that is equivalent to 1 year of clinical use at controlled temperatures (5–55°C) by thermocycler then SBS test by universal testing machine was done. Water contact angle test was done for all specimens per each subgroup (20 specimens) by computer software and an optical tensiometer.

**Results** The SBS was nonsignificantly decreased after thermocycling for all primers embedded with SGO except for ZP primer. The best wettability of ZrO _2_ surface was found in (ZP) primer group embedded with (0.9% SGO) with a mean value of 20.60.

**Conclusion** Primers embedded with SGO could increase the wettability of the ZrO _2_ surface and bond strength durability between resin cement and ZrO _2_ even after thermocycling aging. The clinical significance of this study was the possible increase of the wettability of ZrO _2_ surface and SBS of resin cement to ZrO _2_ with promising long-term stability when commercial primers embedded with SGO were used. This could reduce the risk of debonding between resin cement and ZrO _2_ crowns or veneers.

## Introduction

Zirconia (ZrO _2_ ) has been used in dental restoration due to its excellent mechanical properties, biocompatibility, low degree of bacterial adhesion, and acceptable optical properties, so it is commonly used for crowns and veneers in the anterior teeth. 1 In comparison to other ceramics, Yttria-partially stabilized tetragonal ZrO _2_ polycrystalline restorations have higher mechanical properties. 2 3 One of the major drawbacks for ZrO _2_ is a short-term bond durability with resin cement. This is because the surface of ZrO _2_ is resistant to acids and does not contain silica. 4 Different ZrO _2_ primers used to solve this drawback by enhancing the chemical adherence between ZrO _2_ and resin cement. Previous studies demonstrated that ZrO _2_ surface primers containing 10-methacryloxydecyl dihydrogen phosphate (MDP) and phosphate monomers are reliable chemical agents for improving the bond between ZrO _2_ and resin cement. 5 It can result in a strong bond between ZrO _2_ and resin cement but for a short duration. 6 Graphene oxide (GO) can enhance the mechanical, chemical, and thermal stability of ZrO _2_ by forming covalent bonds with the primer because epoxide and hydroxyl groups are present mainly on its edges. GO is a single-layered sheet of carbon atoms with a two-dimensional hexagonal lattice arrangement that is derived from graphene. 7 The silanization of the GO (silanized nanographene oxide [SGO]) is a very important process which can improve the properties of GO, thus enhancing interfacial interactions with the primer. 8 9 The mechanical properties of the primer can be improved by combining it with nanoscale filler particles. 10 11 Khan et al showed that incorporation of single-walled carbon nanotubes might significantly enhance the resin cement adhesion to ZrO _2_ . 12 Thus, the aim of this study was to evaluate the effect of different primers embedded with (SGO) sheets on wettability of ZrO _2_ surface and the bond strength durability of resin cement to ZrO _2_ .

Therefore, the null hypothesis in the present study was: There was a nonsignificant difference in all primer groups whether added or nonadded SGO regarding the shear bond strength (SBS) testing and water contact angle testing.

## Materials and Methods

### Materials

#### Sample Size Analysis

Sample size calculation was based on mean SBS between different primers retrieved from previous research (Khan et al, 2019). Using G power program version 3.1.9.7 to calculate sample size based on the effect size of 1.06, using the two-tailed test, α error = 0.05 and power = 90.0%, the total calculated sample size will be 20 in each subgroup at least. 13

#### Preparation of Nanographene Oxide

In an ice bath, a 9:1 (360:40 mL) mixture of concentrated sulfuric acid and phosphoric acid (H _2_ SO _4_ /H _3_ PO _4_ ) was first mixed with 3.0 g of native graphite powder to obtain fully oxidized graphene sheets with large lateral sizes. The components were heated to 50°C and stirred for 12 hours. After the reaction reached room temperature, 400 mL of deionized water with 3 mL of 30% hydrogen peroxide was put on ice to separate the GO sheets. Then potassium permanganate is added to the reaction very slowly. After 24 hours at room temperature, a filtered brown GO paste is formed by precipitating the mixture after adding a 10% hydrochloric acid solution. To obtain GO powder, dehydration was performed under vacuum for 6 hours at 60°C. To create nanographene oxide (nGO) sheets, the GO powder was ultrasonically disseminated in water. The resulting brown dispersion was centrifuged at 4,000 rpm for 30 minutes to remove any unexfoliated nGO sheets. 14

#### Preparation of Silanized Graphene Oxide

Briefly, 0.5 g of nGO were mixed with 25 mL ethanol and 25 mL deionized H _2_ O and then dispersed using ultrasonication. This was followed by the addition of 2 mL of vinyltrimethoxysilane (VTMS) and then the mixture was sonicated for 30 minutes then 5 μL of ammonia solution were added. Then using an orbital shaker, the resulting suspension was shaken well for 3 hours, then the supernatant solution was decanted. The obtained precipitate was placed in a hot air oven for 3 hours to be heated at a temperature of 60°C, then the pH was adjusted by repeatedly washing with ethanol and water. Then the precipitate was dried at room temperature to obtain SGO. SGO was prepared according to the method described by Zhi et al. 15

#### Preparation of Zirconia Specimens

Four hundred cube-shaped specimens of presintered 3 mol% ZrO _2_ each measuring 10 mm in width and length and 3 mm in height were milled using milling machine (Cori Tech 350i 5-axis, Germany). The surface was wet-ground with 600-grit silicon carbide abrasive paper, cleaned in a 99.7% ethanol solution for 5 minutes in an ultrasonicator, and then air-dried. Next, the specimens were sandblasted by a blasting device with 110 μm Al _2_ O _3_ particles from a distance of 10 mm at a pressure of 3.5 bar for 15 seconds. Then, for 10 minutes, the specimens were cleansed using an ethanol solution with 99.7% purity in an ultrasonicator and air-dried. Each specimen was embedded in chemical-cured acrylic resin such that the upper surface of the specimen was flushing with that of the acrylic resin. 16

#### Preparation of Primers with SGO

SGO sheets in different concentrations (0.1, 0.3, 0.6, 0.9%) were blended into 1 g of primer. This mix was sonicated for 10 minutes in an ultrasonic bath to produce a uniform solution prior to coating the ZrO _2_ surface.

#### Preparation of the Cement Specimens

A cylindrical cement specimen was created using a rubber tube (3 mm) in diameter on each ZrO _2_ specimen. After positioning the rubber tube over the ZrO _2_ specimen, self-adhesive resin cement (Bisco, Inc., Schaumburg, Illinois, United States) was injected within and cured for 20 seconds by light cure (3M ESPE, Elipar, Deep Cure-L, Germany) according to the manufacturer's instructions of intensity (1,200 mW/cm ^2^ ) output. 17

#### Characterization

The selected samples were scanned using scanning electron microscope (SEM) (TESCAN, VEGA3, Czech Republic) to show the distribution particles of SGO into the primer on the ZrO2 surface.

#### Structural Characterization

The grafting of VTMS on GO monolayer was evaluated and collected in the frequency range of 400 to 4,000 cm ^−1^ by using platinum attenuated total reflection unit attached to Fourier transform infrared spectroscopy (FTIR) spectrometer (Bruker VERTEX 80v FTIR spectrometer, United States) to confirm the covalent bond between silane coupling agent (VTMS) and graphene surface.

#### Morphological Characterization

Transmission electron microscopy (TEM) (JEOL 1200, Japan) was used to investigate the morphology of GO and SGO before and after silanization with VTMS and to show the exfoliated sheets of SGO.

TEM was operated at an accelerated voltage of 200 kV and a spot size of 3. Lateral size distribution was measured using (Image J software version 1.54) (Rasband, 1997–2018) on the acquired TEM images.

### Specimens' Grouping

A total of 400 ZrO _2_ specimens were split into four major groups ( *n* = 100) at random based on the type of primers used:

- Rely X ceramic primer (RX) (3M ESPE, St Paul, Minnesota, United States)
- Monobond N primer (MN) (Ivoclar Vivadent, Schaan, Liechtenstein)
- Monobond plus primer (MP) (Ivoclar Vivadent)
- Z prime plus primer (ZP) (Bisco, Inc., Schaumburg, Illinois, United States).

Each main group was divided into five subgroups of (20 samples) in each subgroup depending on the concentration of SGO (0, 0.1, 0.3, 0.6, and 0.9% wt.).

#### Shear Bond Strength Test

A universal testing machine (Model 3345, Instron, England) was used to measure the immediate SBS between resin cement and ZrO _2_ on half of the specimens in each subgroup (10 specimens). Specimens were positioned vertically and parallel to a round-notched blade in a custom-made specimen holder.

A proprietary software (BlueHil Universal Instron, England) was used to record and collect the data of SBS in an MPa unit at a crosshead speed of 1 mm/min ^−1^ ) until the specimens failed.

The force required to remove the restorative material was measured in Newtons (N) (1 MPa = 1 N/mm ^2^ ), and the SBS value was then calculated in the computer software by dividing the peak load values over the restorative material base area (3.14 mm ^2^ ). 18

#### Thermocycling

The other half of the specimens in each subgroup (10 specimens) were first exposed to thermocycling for 10,000 cycles that is equivalent to 1 year of clinical use at controlled temperatures (5–55°C) by Thermocycler (SD-Mechatronik, GmbH, Germany) to investigate the effect of the thermocycling aging on the bond strength between resin cement and ZrO _2_ , then these specimens exposed to a universal testing machine (Model 3345, Instron, England) to measure the SBS between resin cement and ZrO _2_ .

#### Failure Mode Analysis

Fractured surfaces were observed immediately after SBS test by SEM (TESCAN, VEGA3) of (2.0 kV) at magnification of (×50). The fractured surfaces were classified according to previous work. 13 Adhesive failure was observed when 0 to ≤33% of the resin cement remained on the ZrO _2_ surface. Cohesive failure was observed when >66% but ≤100% of the resin cement remained on the ZrO _2_ surface and mixed failure was observed when >33% but ≤66% of the resin cement remained on the ZrO _2_ surface.

#### Water Contact Angle Test

This test was used to measure the wettability of the ZrO _2_ surface. A 4.0-µL drop of deionized water was applied on (20 specimens) per each subgroup. A digital camera took a picture of the lighted tiny drop from the other side. The software linked to a computer and an optical tensiometer (Theta Lite; Dyne Technology, Lichfield, UK) were used. The mean water contact angle on all ZrO _2_ surfaces were determined after 30 seconds.

### Statistical Analysis

Numerical data were shown as mean and standard deviation (SD) values. They were explored for normality by checking the data distribution and using Shapiro–Wilk's test. Data showed parametric distribution. One-way analysis of variance (ANOVA) followed by Tukey's post hoc test was used for intergroup and intragroup comparisons to analyze SBS and the water contact angle tests ( Table 1 ).

**Table 1:** Materials used in the study, description, manufacturer, lot number, and its composition

Materials	Description	Manufacturer	Lot number	Composition
Yttrium-stabilized zirconia (Y _2_ O _3_ ).	High translucent Zirconia-based ceramics (14 mm) Presented as tetragonal crystal phase partially stabilized	Katana, Japan		3% mol of yttrium oxide, ZrO _2_
Z prime plus primer	Adhesive primer	Bisco, Inc., Schaumburg, Illinois, United States	2300010396	Ethanol alcohol, MDP, BPDMA, HEMA
Monobond N primer	Adhesive primer	Ivoclar Vivadent, Schaan, Liechtenstein	X17917	Ethanol, water, acetic acid, 3-methacryloxypropyltrimethoxysilane
Monobond plus primer (universal primer)	Adhesive primer	Ivoclar Vivadent, Schaan, Liechtenstein	Y35161	Ethanol, water, silane methacrylate, methacrylated phosphoric acid ester, sulphide methacrylate, 3-trimethoxysilyl propyl methacrylate MDP
Rely X primer	Adhesive primer	3M ESPE, St Paul, Minnesota, United States	NF20769	Ethanol, water, 3-methacryloxypropyltrimethoxysilane
BisCem	Self-adhesive resin cement-dual cured	Bisco, Inc., Schaumburg, Illinois, United States	2100004807	Base: bisphenol-A glycidyl dimethacrylate, uncured dimethacrylate monomer, glass fillers Catalyst: phosphate acidic monomer, glass fillers

Abbreviations: BPDMA, aromatic substituted carboxylic acid monomer dimethacrylate; HEMA, 2-hydroxyethyl methacrylate; MDP, 10-methacryloxydecyl dihydrogen phosphate.

## Results

### Characterization of GO

#### Scanning Electron Microscope Analysis

It illustrated well-distributed SGO in the primer after spreading on the ZrO _2_ surface with a lot of aggregates of SGO particles appeared as cauliflower and particles of needle-shaped in between.

#### FTIR

The FTIR spectrum of GO showed peaks specific to exfoliated GO located at 1,732, 1,600, 1,360, 1,054, and 955 cm ^−1^ matching the mode of vibration of the alkoxy (C–O) group. FTIR spectrum of SGO showed new bands appeared in the range of 900 to 1,100 cm ^−1^ corresponding to silicon bonds such as single silicon bonds with oxygen, carbon, and hydroxyl group. It also showed aliphatic carbon double bond of the vinyl functional groups of VTMS at 1,640 cm ^−1^ . 19

#### TEM

**Fig. 1:**
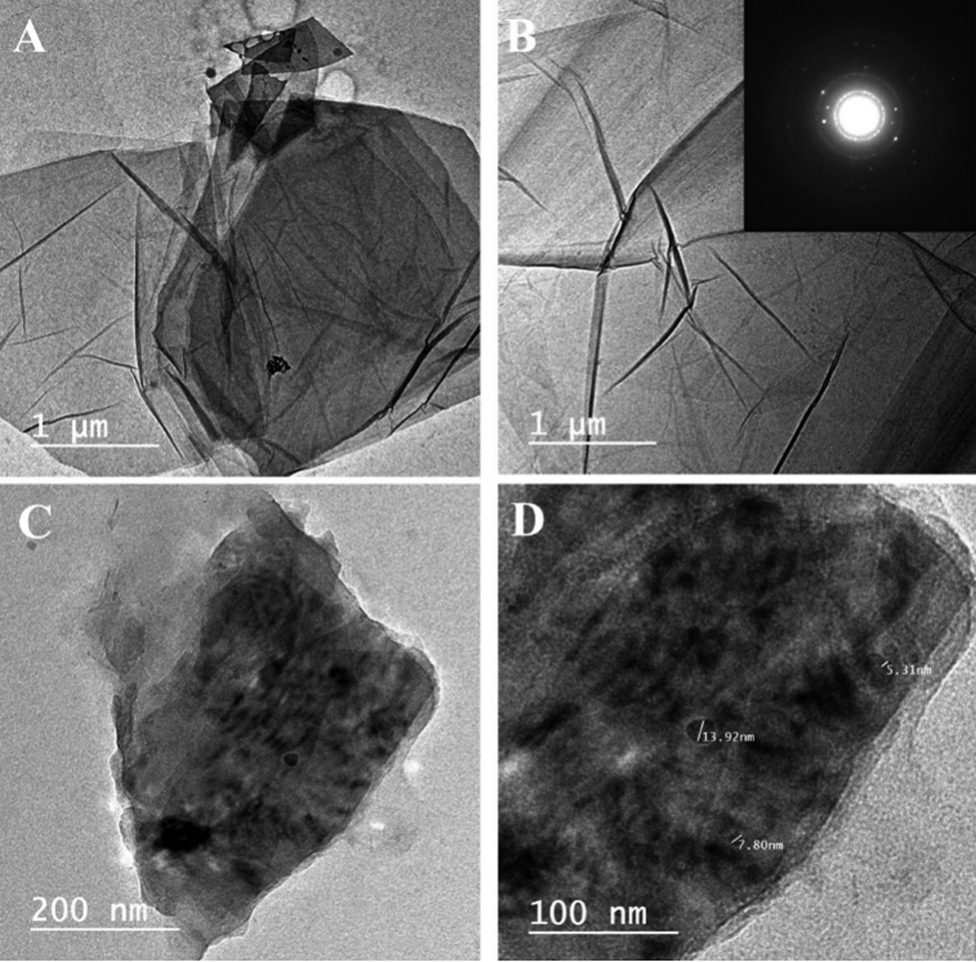
( **A, B** ) Transmission electron microscopy micrographs of graphene oxide and VTMS-grafted graphene oxide (silanized nanographene oxide).

The highly oxidized GO sheets are shown in TEM micrographs in Fig. 1A, B that the structure of GO sheets is smooth and only monolayer of carbon was detected. 20 The sheets of SGO in TEM micrographs shown in Fig. 1C, D was darker as compared with GO sheets. Few silica nanoparticles smaller than 20 nm were detected on the sheet. 15

#### Shear Bond Strength Test

A one-way ANOVA test was used to determine the mean ±  SD, and the post hoc Bonferroni correction was used to compare groups and to compare subgroups multiple times.

The highest immediate SBS mean value was found in the ZP primer group at its (0.9% SGO) subgroup (mean = 30.41 MPa). The lowest SBS mean value after thermocycling was found in the ZP primer group at its control subgroup (mean = 10.62 MPa). For ZP primer group, there was a significantly decreased of SBS after thermocycling at all subgroups. For other primer groups, there was a nonsignificantly decrease of SBS after thermocycling at all subgroups except control subgroup. For immediate SBS column in Table 2 , there was a significant difference among all primer groups at 0.6 and 0.9% SGO subgroups. For SBS after thermocycling column in Table 2 , there was a significant difference among all primer groups at all subgroups.

**Table 2:** Comparison between (Z prime, monobond N, monobond plus, and rely X) primer groups and their subgroups of SGO concentrations according to immediate SBS and SBS after thermocycling ( *n* = 20 specimens per each subgroup, 10 specimens exposed to immediate SBS test and 10 specimens exposed to SBS test after thermocycling)

Subgroups	Groups	Immediate SBS (mean), MPa	SD	SBS after thermocycling (mean), MPa	SD	*p* -Value
0% SGO (control)	Z prime plus	23.68	2.18	10.62 ^A^	2.4	0.000 *
	Monobond N	21.21	0.77	12.23 ^B^	0.24	0.004 *
	Monobond plus	22.56	0.37	10.957 ^C^	0.15	0.000 *
	Rely X	21.56	1.9	15.25 ^ABC^	1.75	0.006 *
1% SGO	Z prime plus	25.77	1.84	13.27 ^ABC^	0.433	0.000 *
	Monobond N	23.66	1.53	19.737 ^AD^	3.85	0.067
	Monobond plus	25.18	3.39	23.21 ^BD^	3.72	0.079
	Rely X	24.08	1.12	21.15 ^C^	2.75	0.254
3% SGO	Z prime plus	27.18	0.05	14.76 ^ABC^	0.67	0.000 *
	Monobond N	26.33	2.33	19.85 ^ADE^	3.46	0.055
	Monobond plus	28.77	0.31	26.93 ^BDF^	3.81	0.063
	Rely X	27.59	1.41	22.89 ^CEF^	2.46	0.078
6% SGO	Z prime plus	29.88 ^A^	5.75	17.26 ^ABC^	0.28	0.000 *
	Monobond N	27.14 ^AB^	2.12	21.25 ^ABDE^	3.16	0.055
	Monobond plus	30.24 ^BC^	1.92	27.44 ^ABD^	3.92	0.082
	Rely X	27.91 ^C^	1.84	22.4 ^ABCE^	3.05	0.123
9% SGO	Z prime plus	30.41 ^A^	1.32	18.45 ^ABC^	1.27	0.000 *
	Monobond N	28.15 ^AB^	1.55	21.65 ^ADE^	4.38	0.073
	Monobond plus	30.34 ^B^	1.56	28.493 ^BD^	4.07	0.097
	Rely X	29.38 ^A^	1.97	23.54 ^CE^	4.1 9	0.094

Abbreviations: SBS, shear bond strength; SD, standard deviation; SGO, silanized nanographene oxide.

Notes: Similar capital letters in the same column denote a significant difference among primer groups. The capital letter “A” represents the *p* -value of Z prime plus and monobond N; “B” represents the *p* -value of Z prime plus and monobond plus; “C” represents the *p* -value of Z prime plus and rely X; “D” represents the *p* -value of monobond N and monobond plus; “E” represents the *p* -value of monobond N and rely X); and “F” represents the *p* -value of monobond plus and rely X primer groups.

* A significance level of *p* -value <0.05.

### Failure Mode Analysis

The examination revealed several types: Adhesive failure occurred at the resin cement and ZrO _2_ interface in the primers without SGO groups. Cohesive failure occurred at ZrO _2_ or resin cement in the groups of primers without SGO. Mixed failure (combination of adhesive and cohesive failures) occurred in the primers without SGO groups.

### Water Contact Angle Test

A one-way ANOVA test was used to determine the mean ± SD, and the post hoc Bonferroni correction was used to compare groups and to compare subgroups. Table 3 shows that the highest mean value of water contact angle was found in the RX primer group at its control subgroup (mean = 44.70). The lowest mean value of water contact angle was found in the ZP primer group at its 0.9% SGO subgroup (mean = 20.60). There was a significant difference among all subgroups of all primer groups ( *p* -value = 0.000 ^*^ ). There was a nonsignificant difference between MN and RX primer groups at 0, 0.1, and 0.3% SGO subgroups only.

**Table 3:** Comparing the groups using the water contact angle (θ) test for the primers: monobond plus, monobond N, rely X, and Z prime plus and their subgroups according to SGO concentrations ( *n* = 20 specimens per each subgroup)

Groups	0% SGO (control) subgroup	0.1% SGO subgroup	0.3% SGO subgroup	0.6% SGO subgroup	0.9% SGO subgroup	*p* -Value
Z prime plus	37.40 ± 1.90 ^a^	26.67 ± 0.51 ^b^	25.70 ± 0.40	22.80 ± 0.82 ^d^	20.60 ± 1.21 ^e^	0.000 *
Monobond N	43.20 ± 2.20 ^aA^	38.67 ± 1.21 ^bA^	36.93 ± 0.60 ^A^	34.83 ± 0.78 ^d^	32.27 ± 0.61 ^e^	0.000 *
Monobond plus	40.30 ± 2.00 ^a^	34.67 ± 0.47 ^b^	31.90 ± 0.56	26.93 ± 0.75 ^d^	25.13 ± 0.91 ^e^	0.000 *
Rely X	44.70 ± 2.20 ^aA^	39.50 ± 0.10 ^bA^	35.50 ± 0.30 ^A^	31.43 ± 0.32 ^d^	29.50 ± 0.46 ^e^	0.000 *
	*p* < 0.001 *	*p* < 0.001 *	*p* < 0.001 *	*p* < 0.001 *	*p* < 0.001 *	

Abbreviation: SGO, silanized nanographene oxide.

Notes: Similar capital letters in the same column denote a nonsignificant difference among the primer groups. Different small letters denote a significant difference among the subgroups. The capital letter “A” represents the *p* -value of Z prime plus and monobond N primer groups.

* A significance level of *p* -value <0.05.

## Discussion

The bond strength between resin cement and ZrO _2_ increases by the presence of MDP in the primers. Moreover, it has been suggested to extend the application of MDP to primers for ZrO _2_ bonding to resin cement. 21

The bond strength between resin cement and ZrO _2_ also increases by sandblasting particularly with Al _2_ O _3_ air abrasion in the presence of primers with MDP which helps increasing the surface energy and wettability of the ZrO _2_ surface. 22

Therefore, SGO sheets were chosen in the present study because of their attractive qualities, as they provide mechanical, chemical, and thermal stability of the ZrO _2_ . The mechanical property was improved by providing a shock absorbing elastic layer between resin cement ZrO _2_ when it is added to the primer. It can also reduce polymerization shrinkage of the primer and form covalent bond with resin matrix because epoxide and hydroxyl groups are present mainly on its edge. 13 Increasing GO up to 1% per weight concentration can increase all mechanical properties. 23

SEM, FTIR, and TEM were used in combination to analyze and characterize GO and SGO chemically.

**Fig. 2:**
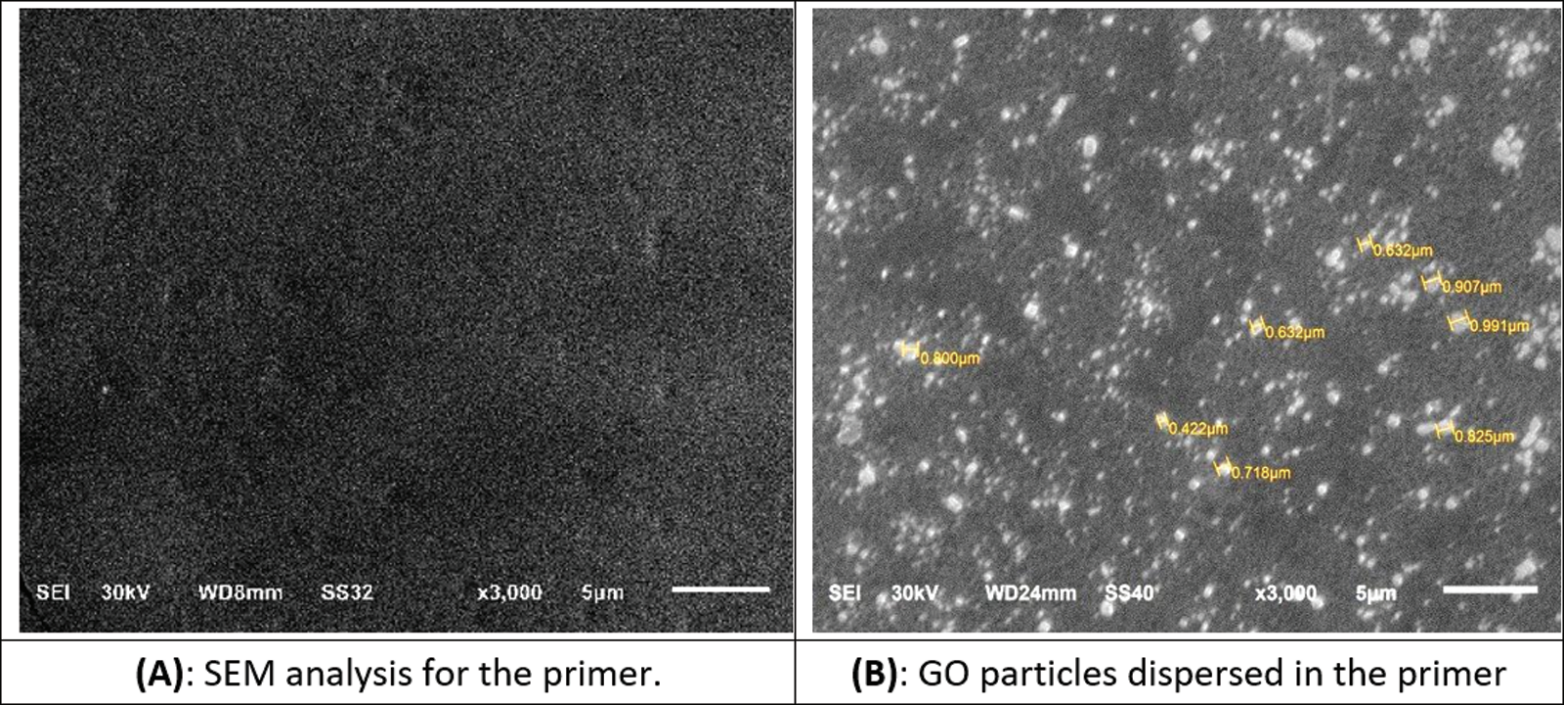
( **A** ) SEM image for the primer structure without SGO and ( **B** ) SEM image for SGO which is well distributed in the primer after spreading on the zirconia surface. SEM, scanning electron microscope; SGO, silanized nanographene oxide.

Regarding SEM, it was used after the SGO was embedded with primers, and it showed well-distributed SGO in the primer after spreading on the ZrO _2_ surface with a lot of aggregates of SGO particles appearing as cauliflower and particles of needle-shaped in between due to the presence of van der Waals forces, which bind the sheets of GO together, as shown in Fig. 2 . These findings were in agreement with those of previous studies. 24 25

**Fig. 3:**
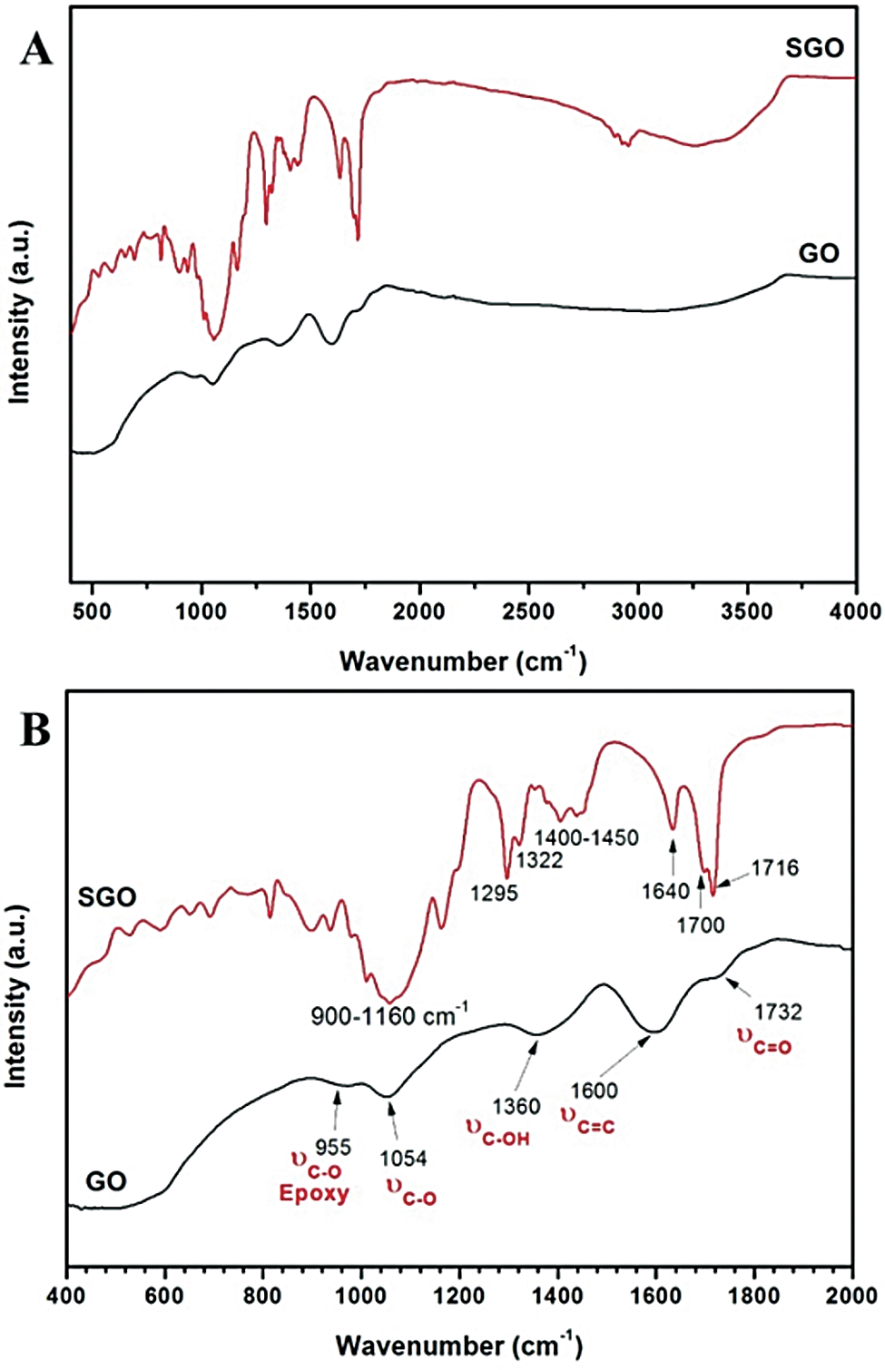
FTIR spectrum of GO (black line) and VTMS-grafted graphene oxide (SGO; red line) within the range of wavenumber 400 to 4,000 cm ^−1^ ( **A** ) and in the wavenumber range of 400 to 2,000 cm ^−1^ ( **B** ). FTIR, Fourier transform infrared spectroscopy; GO, graphene oxide; SGO, silanized nanographene oxide.

Regarding FTIR, to confirm the covalent interaction between silane coupling agent (VTMS) and graphene surface, infrared spectra of GO and SGO were collected as shown in Fig. 3 . The FTIR spectrum of GO has specific peaks to exfoliated GO located at 1,732, 1,600, 1,360, 1,054, and 955 cm ^−1^ attributed to (carbonyl (carbon double bond Oxygen), aromatics (carbon double bond), carbon single bond hydroxyl group and epoxy groups (carbon single bond oxygen) respectively). In the case of SGO spectrum, new bands appeared in the range of 900 of 1,100 cm ^−1^ attributed to silicon bonds (such as single silicon bonds with oxygen, carbon, hydroxyl group) originated from VTMS structure or the formation of silica nanoparticles after silanization. Aliphatic carbon double bond of the vinyl functional groups of VTMS was detected at 1,640 cm ^−1^ , indicating the availability of the vinyl groups on the surface of graphene to covalently link with the primers used in this study. 19

The highly oxidized GO sheets are shown in TEM micrographs in Fig. 1A, B . It is evident that the structure of GO sheets is smooth and only monolayer of carbon was detected due to the exfoliation of bulk graphite oxide due to the comparatively high number of oxygenated functional groups that are present on the edges and surface of GO sheets. The sheets of SGO in TEM micrographs shown in Fig. 1C, D were darker as compared with GO sheets due to the silanization with VTMS. Few silica nanoparticles smaller than 20 nm were detected on the sheet due to the hydrolysis and condensation reaction of silane coupling agents that might occur between silanol groups of nongrafted VTMS. These results were in agreement with previous studies. 15 20 Higher crystallinity was clearly evident in these new structures where bright areas corresponded to crystalline structures sharing a homogenous orientation. Higher resolution TEM not only showed this increased crystallinity but also showed graphene domains. 26

Regarding the water contact angle testing, measurement of contact angle provides a quick and useful method to investigate surface wettability of ZrO _2_ surface by tensiometer. Primers increase the surface wettability and allow a better flow of resin cement on the ZrO _2_ surface. In the present study, the contact angle values were less than 45 degrees in all primer groups embedded with SGO at all concentrations indicating adequate wetting of the ZrO _2_ surface. When SGO concentrations increased, the water contact angle decreased in all primer groups. This could be due to that SGO is characterized by hydrophilicity due to the presence of carbonyl and carboxyl groups at its edges and oxygen-containing functional groups on its basal plane which suggested a good wetting of ZrO _2_ surface and strong connection of resin cement to ZrO _2_ .

ZP primer group with (0.9% SGO) had the lowest mean value among all primer groups (20.60). This could be due to the presence of SGO and hydroxyethyl methacrylate (HEMA) content in its composition which enhanced hydrophilicity and decreased water removal at the ZrO _2_ surface. RX primer control group had the greatest mean value among all primer groups (44.70). This could be due to the absence of GO in this primer's control group. These results were in agreement with previous studies. 27 28 There was a nonsignificant difference between MN and RX primer groups at 0.1, 0.3, and 0.6% SGO only, this could be due to these two primers embedded with SGO had similar compositions of ethanol, water, 3-methacryloxypropyltrimethoxysilane.

Regarding SBS testing, it is the most commonly used, easiest, and fastest method to analyze the significant influence of various parameters of ZrO _2_ surface bonding to the resin cement. For immediate SBS column in Table 2 , there was a significant difference among all primer groups at 0.6 and 0.9% SGO subgroups. This could be due to the effect of increasing SGO on the weight of different elements of the primers which may affect the SBS values. 13 For SBS after thermocycling column in Table 2 , there was a significant difference among all primer groups at all subgroups. This could be due to the effect of SGO and thermocycling aging by bond hydrolysis and thermal stresses on the weight of different elements of the primers which may affect the SBS values.

ZP primer group with 0.9% SGO had the highest SBS mean value among all primer groups (30.41 MPa) before thermocycling. This is because the effectiveness of MDP and SGO is increasing the chemical bond between resin cement and ZrO _2_ . ZP primer control group had the lowest SBS mean value among all primer groups (10.62 MPa) after thermocycling. This could be due to the absence of SGO which can increase the SBS value. 29

This study showed that the SBS between resin cement and ZrO _2_ was slightly decreased after thermocycling for 10,000 cycles, which is equivalent to 1 year of clinical use. The bond strength decreases with increasing number of thermocycles due to the increased water absorption. 28

In the present study, SGO embedded with primers increased the mechanical strength by offering an elastic layer capable of shock absorption between the resin cement and the ZrO _2_ and by withstanding the stresses of thermocycling thanks to the mechanical, chemical, and thermal stability provided by the SGO. SGO had amphiphilic nature permitted water to move from hydrophilic to hydrophobic carbon sites and decreased the effects of thermocycling aging on the adhesive layer and the interface between resin cement and ZrO _2_ . 29

For RX and MN primer groups embedded with all SGO concentrations subgroups, there was a nonsignificantly decrease in SBS after thermocycling compared with its immediate SBS. This provided SBS stability and durability of resin cement to ZrO _2_ . It could be due to the presence of 3-methacryloxypropyltrimethoxysilane in its composition which enhanced the resin's tensile modulus, water resistance, thermal stability, and bond strength between resin cement and ZrO _2_ . For MP primer group embedded with all SGO concentrations, there was a nonsignificantly decrease in SBS after thermocycling compared with its immediate SBS. This provided SBS stability and durability of resin cement to ZrO _2_ . It could be due to the effectiveness of MDP and SGO in increasing the bond strength between ZrO _2_ and resin cement. These results were in agreement with previous studies. 13 30

For ZP primer group, there was a significantly decrease in SBS after thermocycling compared with its immediate SBS. This reduced the SBS stability and durability of resin cement to ZrO _2_ . This could be due to thermal stresses and bond hydrolysis as a result of thermocycling aging which caused adhesive failures and then reduced the bond strength between resin cement and ZrO _2_ . 31 Also, it could be due to the presence of HEMA in its composition which increased hydrophilicity, decreased water removal, and decreased copolymerization that may decrease the bond strength between resin cement and ZrO _2_ . 25

**Fig. 4:**
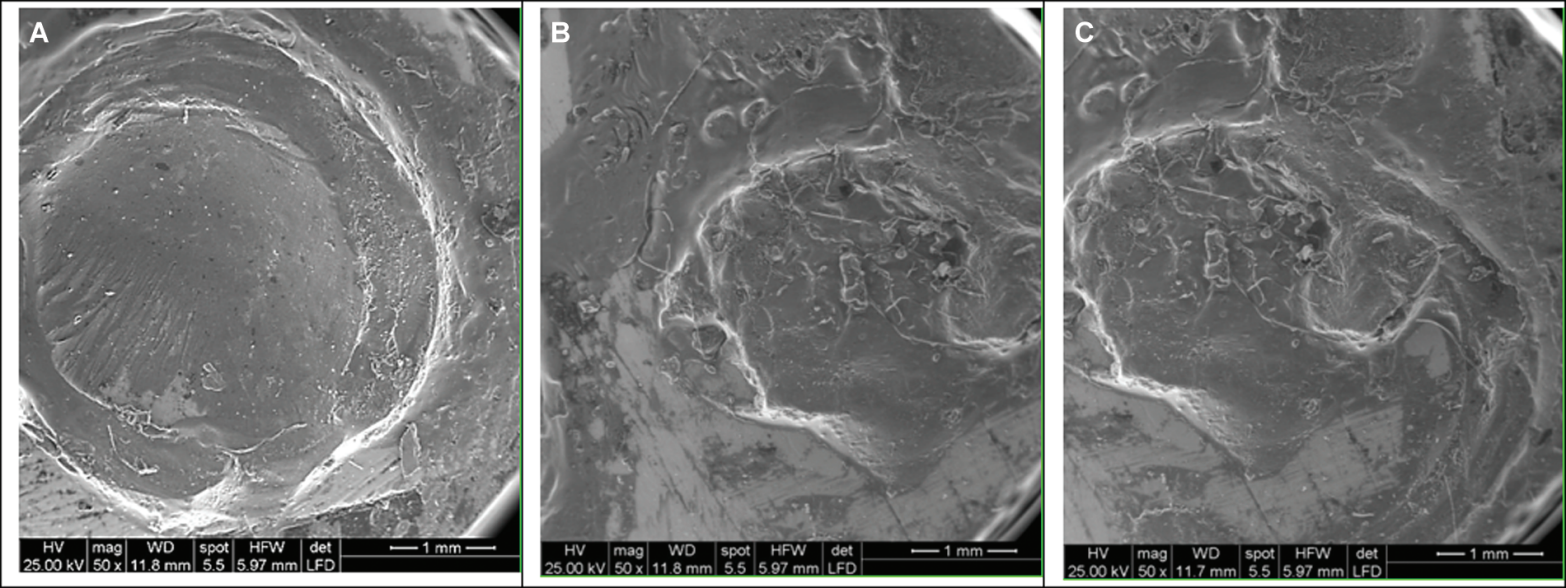
SEM images of ZrO _2_ surface showing different modes of failure. ( **A** ) Adhesive failure at the resin cement and ZrO _2_ interface. ( **B** ) Cohesive failure at ZrO _2_ or resin cement. ( **C** ) Mixed failure (combination of cohesive and adhesive failures).

Regarding failure mode analysis, it was observed in SEM analysis, mixed and cohesive fractures increased in the SGO embedded with primer groups which improved the durable bond strength of the ZrO _2_ to resin cement. Primers without SGO mainly exhibited adhesive failure mode which indicates the weak bond strength between the ZrO _2_ and resin cement interface. These results were in agreement with previous studies ( Fig. 4 ). 13

Depending on the results of the present study, the null hypothesis of this study was rejected as SBS between resin cement and ZrO _2_ was improved when SGO was blended with all primers even after thermocycling. The water contact angle of the ZrO _2_ surface was improved in all primer groups when SGO was blended with it.

The limitations of the present study are as follows: (1) There was only one commercial kind of resin cement covered in this study. (2) Results of this study were difficult to compare with previous studies due to the difference in methodologies applied such as different aging techniques and the limited available data in the literature concerning this topic.

The clinical significance of this study was the possible improvement of wettability of ZrO _2_ surface and SBS of resin cement to ZrO _2_ with promising long-term stability when commercial primers embedded with SGO were used. This could reduce the risk of debonding between resin cement and ZrO _2_ crowns or veneers.

Using different commercial types of adhesive cements is recommended in future studies. Further mechanical testing for bonding between adhesive cements and ZrO _2_ is also recommended with investigations after different aging conditions to assess the validity of SBS.

## Conclusion

According to the limitations of this study and the data obtained, the primers embedded with SGO could improve the wettability of the ZrO _2_ surface and bond strength durability between resin cement and ZrO _2_ even after thermocycling aging.
